# Mobile Apps for Dietary and Food Timing Assessment: Evaluation for Use in Clinical Research

**DOI:** 10.2196/35858

**Published:** 2023-06-16

**Authors:** Siena Gioia, Irma M Vlasac, Demsina Babazadeh, Noah L Fryou, Elizabeth Do, Jessica Love, Rebecca Robbins, Hassan S Dashti, Jacqueline M Lane

**Affiliations:** 1 Center for Genomic Medicine Massachusetts General Hospital Boston, MA United States; 2 Division of Sleep and Circadian Disorders Brigham and Women’s Hospital Boston, MA United States; 3 Medical and Population Genetics Program Broad Institute Cambridge, MA United States; 4 Division of Sleep Medicine Harvard Medical School Boston, MA United States; 5 Department of Anesthesia, Critical Care and Pain Medicine Massachusetts General Hospital Boston, MA United States

**Keywords:** dietary assessment, mobile phone, smartphone, nutrition apps, dietary record, circadian rhythms, food diary, food timing

## Abstract

**Background:**

Over the last decade, health mobile apps have become an increasingly popular tool used by clinicians and researchers to track food consumption and exercise. However, many consumer apps lack the technological features for facilitating the capture of critical food timing details.

**Objective:**

This study aimed to introduce users to 11 apps from US app stores that recorded both dietary intake and food timing to establish which one would be the most appropriate for clinical research.

**Methods:**

To determine a viable app that recorded both dietary intake and food timing for use in a food timing–related clinical study, we evaluated the time stamp data, usability, privacy policies, the accuracy of nutrient estimates, and general features of 11 mobile apps for dietary assessment that were available on US app stores. The following apps were selected using a keyword search of related terms and reviewed: text entry apps—Cronometer, DiaryNutrition, DietDiary, FoodDiary, Macros, and MyPlate; image entry apps—FoodView and MealLogger; and text plus image entry apps—Bitesnap, myCircadianClock, and MyFitnessPal.

**Results:**

Our primary goal was to identify apps that recorded food time stamps, which 8 (73%) of the 11 reviewed apps did. Of the 11 apps, only 4 (36%) allowed users to edit the time stamps. Next, we sought to evaluate the usability of the apps using the System Usability Scale across 2 days, and 82% (9/11) of the apps received favorable scores for usability. To enable use in research and clinical settings, the privacy policies of each app were systematically reviewed using common criteria, with 1 (9%) Health Insurance Portability and Accountability Act–compliant app (Cronometer). Furthermore, protected health information was collected by 9 (82%) of the 11 apps. Finally, to assess the accuracy of the nutrient estimates generated by these apps, we selected 4 sample food items and a 3-day dietary record to input into each app. The caloric and macronutrient estimates of the apps were compared with the nutrient estimates provided by a registered dietitian using the Nutrition Data System for Research database. In terms of the 3-day food record, the apps were found to consistently underestimate daily calories and macronutrients compared with the Nutrition Data System for Research output.

**Conclusions:**

Overall, we found that the Bitesnap app provided flexible dietary and food timing functionality capable of being used in research and clinical settings, whereas most other apps lacked in the necessary food timing functionality or user privacy.

## Introduction

### Background

Smartphone use around the globe has increased tremendously in the past 2 decades, with 85% of Americans possessing a smart device in 2021 [1]. Traditionally, dietary assessment methods use paper-based food diaries (FDs); however, the emergence of mobile technologies and prevalence of smartphone ownership position smartphones as innovative tools for real-time dietary assessments. Dietary assessment is an important component of clinical studies that often poses a challenge to researchers, particularly in studies exploring food timing. Recently, food timing has become a focus of research owing to emerging evidence of the metabolic effects of the timing of food consumption and intermittent fasting in humans [2,3]. Interventional studies have observed that participants with high caloric intake in the morning have greater weight loss and lower glucose and insulin levels compared with those with high caloric intake in the evening [4]. Similarly, several observational studies have found associations between evening dietary intake and obesity [5-7], and interventional studies have found that evening mealtime increases glucose intolerance [8,9]. These findings support a growing body of research suggesting that the timing of food consumption is a critical factor of cardiometabolic health, body weight regulation, and metabolism in humans.

The circadian system is a network of molecular clocks that produces circadian rhythms, which are physiological and behavioral processes. Extrinsic time cues (known as zeitgebers) can influence circadian clocks and lead to internal desynchrony [10,11]. Emerging studies suggest that food consumption time is a zeitgeber in peripheral clocks that can contribute to circadian rhythm desynchronization, which may contribute to adverse physical and psychiatric health effects [10,12,13]. Further investigation is needed to explore how food timing impacts the human circadian system and overall health. However, the absence of standard dietary assessment methods that capture food timing is a major barrier to this field of research. Currently, dietary intake is assessed using a multitude of methods, including food frequency questionnaires; 24-hour dietary recalls (24HRs); and food records, also known as FDs [14]. Food frequency questionnaires require respondents to indicate the frequency of food and beverage consumption and the amount of food and beverages consumed over time, typically a calendar year; however, these assessments do not capture the timing of food intake [14]. In 24HRs, respondents list all foods and beverages consumed in the preceding 24 hours [14]. A major limitation of 24HRs is its reliance on the respondents’ memory of food consumption and timing [14]. By contrast, FDs require respondents to record food and beverage intake at the time of consumption and does not rely on the respondent’s memory; thus, food timing may be recorded more accurately [14]. However, FDs have several limitations, including literacy requirements and a high participant burden [14]. Recently, researchers have explored electronic FDs as a method for reducing burden and increasing the validity of data, but digital privacy presents concerns for participants and researchers alike.

### Benefits and Challenges

There are numerous benefits of electronic FDs: their implementation reduces participant burden; standardizes data collection; improves data quality; and lowers costs, particularly when photo entry and accurate automated nutrient calculations are used [15-18]. Moreover, studies have shown a patient preference for electronic methods, in addition to greater user satisfaction and adherence, indicating that the use of mobile apps in dietary assessment can greatly benefit both participants and researchers [19-21]. Mobile health (mHealth) apps are becoming a vital aspect of scientific research, as they offer researchers the ability to track food intake, sleep cycles, exercise, and more. The widespread availability of smartphones makes mobile apps a promising method that is scalable and facilitates real-time dietary assessment. Accordingly, mobile apps have become an increasingly popular tool in clinical practice in recent years, with them becoming the most widely used technology in registered trials [21]. Despite the benefits of mHealth apps for dietary assessment, there are several challenges to incorporating these technologies into clinical practice and research [22]. These challenges include data privacy [23], security concerns, and data collection without intervention or user feedback. Moreover, 78% of Americans have reported concerns regarding the government or corporations tracking their devices and behavior [24]. In addition, as data breaches have increased by almost 50% over the past decade, with 1001 public data breaches in 2020 alone, the security of personal information, including health information, is a valid concern expressed by both participants and researchers [25].

Current legislation either lacks proper policies or is too outdated to adequately respond to the functionality of mHealth apps, and lawmakers and regulators in the United States and around the world have been slow in passing and implementing digital privacy protection policies [26]. To market their mHealth apps on both Apple iOS (Apple Inc) and Google Play (Google LLC), companies with apps need to maintain, distribute, and present a privacy policy.

However, most individuals have a poor understanding of privacy policies. Consequently, people most often neglect to read the privacy policies provided [24]. Laws intended to protect individuals’ health privacy, notably the Health Insurance Portability and Accountability Act (HIPAA) passed in 1996, do not provide the requirements pertaining to the storage and distribution of health information on digital sites in distinct and clear language. Most privacy policies of mHealth apps are vague and basic and fail to adhere to the simple and limited requirements of HIPAA, creating a major limitation for researchers and clinicians hoping to incorporate innovative mobile tools.

Another major challenge in the use of mobile technology in clinical practice is app selection. As of 2017, over 350,000 mHealth apps were available on Android and Apple iOS app stores [22]. Currently, there is no standard mobile app for dietary assessment, and the sizable number of health apps available makes app selection a considerable challenge for researchers. To our knowledge, the usability and validity of food tracking mobile apps in the context of clinical research, particularly in studies of circadian rhythms and food timing in populations with shifted mealtimes, have not been evaluated. Given the increased prevalence of metabolic disorders and adverse health implications associated with delayed meal timing [27-30], future clinical investigation is needed to explore how meal timing influences overall health and the circadian system. Therefore, efficient and scalable dietary assessment methods that accurately capture food timing are necessary. Mobile apps are a promising tool for dietary assessment with the potential to greatly improve efficiency if photo diaries are coupled with artificial intelligence (AI) identification and quantification of food logs [18,31-34]. Thus, we focused on evaluating the usability, food timing features, data privacy and security, and accuracy of nutrient estimates while assessing the apps. Given that no standard dietary assessment app exists, we evaluated several apps and determined that our evaluation would be beneficial to researchers and clinicians seeking to adopt mobile apps for dietary assessment in clinical and research practice.

## Methods

### Selection of Mobile Apps for Dietary Assessment

Mobile apps for dietary assessment were identified using a keyword search of the terms *food diary*, *food view*, *meal logger*, *food log*, and *diary of nutrition* in the Apple App Store and Google Play Store in November 2020. Apps requiring purchase, apps with ratings <2 stars, and apps with <50 downloads were excluded from the review. On the basis of these criteria, 9 apps were selected. Furthermore, 2 additional apps, MealLogger and myCircadianClock, were included, as they have been used in prior circadian rhythm research (Table 1). The 11 apps selected for evaluation were Bitesnap, Cronometer, DiaryNutrition, DietDiary, FoodDiary, FoodView, Macros, MealLogger, MyPlate, myCircadianClock, and MyFitnessPal. Of the 11 apps, 8 (73%) were available on both iOS and Android devices, and 3 (27%) were only available on Android. The following apps permitted text entries only: Cronometer, DiaryNutrition, DietDiary, FoodDiary, Macros, and MyPlate. The following apps permitted image entries only: FoodView and MealLogger. The following apps permitted both text and image entries: Bitesnap, myCircadianClock, and MyFitnessPal.

**Table 1:** User reviews and ratings of the selected 11 food logging mobile apps as of February 18, 2021.

App names	iOS	Apple App Store ratings (0-5)	App store reviews, n	Android	Google Play Store ratings (0-5)	Google Play reviews, n	Installs (Android^a^)
Bitesnap^b^	✓	4.8	1095	✓	4.7	2949	>100,000
Cronometer^c^	✓	4.8	18,818	✓	4.5	12,035	>1 million
DiaryNutrition		N/A^d^	N/A	✓	4.4	2068	>100,000
DietDiary		N/A	N/A	✓	4.7	1111	>50,000
FoodDiary		N/A	N/A	✓	4.2	3485	>100,000
FoodView	✓	5.0	2	✓	4.4	67	>10,000
Macros	✓	4.6	116	✓	4.5	8510	>1 million
MealLogger	✓	3.3	9	✓	3.3	235	>50,000
MyPlate	✓	4.6	26,633	✓	4.5	40,715	>1 million
myCircadianClock	✓	2.2	160	✓	2.5	289	>50,000
MyFitnessPal	✓	4.7	1,181,099	✓	4.5	2,391,449	>50 million

^a^No install statistics were available for iOS.

^b^Developed in 2016.

^c^Developed in 2005.

^d^N/A: not applicable.

### App Evaluation

Each mobile app was evaluated using the System Usability Scale (SUS), a 10-item scale developed by John Brooke that has become the industry standard for determining the usability of various products, including mobile apps [35]. To assess usability, users were asked to rate 10 statements involving several aspects of usability, including complexity, ease of use, functionality, and consistency, on a 5-point Likert scale ranging from “strongly agree” to “strongly disagree” [35]. The SUS scores range between 1 and 100, with a score >68 considered above average [35]. To assess the usability of the mobile apps for dietary assessment selected for review, 7 users downloaded and evaluated each app using the SUS after 2 days. Of these 7 users, 4 (57%) used iOS smartphones, and 3 (43%) used Android smartphones. The testing group consisted of 2 researchers, 2 members of the lay public, and 3 students and staff who were naive to the research. The mean SUS score for each app was calculated.

In addition to usability, data privacy and security were assessed by systematically reviewing the apps’ privacy policies against the set criteria created by the researchers, which focused on the apps’ collection, transmission, and storage of user data. We identified and reviewed the features present in the mobile apps for dietary assessment that are of interest to researchers when selecting an app for clinical use. These features include data input method, data tracked, food timing features, food composition database (FCD), and feedback to users.

The accuracy of the nutrient estimates of the mobile apps for dietary assessment was assessed using 4 sample food items. The 4 food items (a medium orange, 3 Oreo cookies, a peanut butter and jelly sandwich, and a Burger King Whopper [Burger King Corp]) were selected as examples of a generic fruit, branded item, homemade food, and fast-food meal, respectively. One of the researchers entered the 4 food items into each app, and a registered dietitian entered the 4 food items into the Nutrition Data System for Research (NDSR) database to estimate caloric and macronutrient content. The caloric, carbohydrate, fat, and protein estimates of each item from each app and NDSR were recorded. The relative difference of caloric and macronutrient estimates between the apps and the NDSR reference were computed, and a paired 2-tailed *t* test was performed to determine potential statistical significances.

The nutrient estimates of the apps that estimated nutrient values (Bitesnap, Cronometer, Macros, MyPlate, and MyFitnessPal) were compared with the estimates from the NDSR reference using a 3-day food record. The 3-day food record used for analysis was completed by a single researcher on 3 consecutive days, consisting of 2 weekdays and 1 weekend day. One of the researchers logged all the food items from the 3-day record and the 4 sample food items into the 5 apps in a consistent manner. Each food item was identified using a text search, and the first result was selected. Identical portion sizes were selected for each food item in each of the apps. In apps that specified foods that have been “verified,” indicating that their nutritional information has been reviewed, the first verified search result was selected for each food item. Nutrient estimates from the NDSR database were determined by a registered dietitian nutritionist who was provided with the same 4 sample food items and 3-day food record. Finally, we compared the macronutrient estimates of the apps with those of NDSR using a 1-way ANOVA.

### Statistical Analysis

Relative difference was calculated as follows: *([app reference] / reference) × 100*. The apps’ nutrient estimate accuracy for the 4 sample food items and the 3-day food record was determined by comparing the mean caloric and macronutrient estimates between the apps and the NDSR reference using a paired 2-tailed *t* test. Differences in the nutrient estimates given by the 3-day food record were additionally assessed using 1-way ANOVA. All statistical analyses were performed using R (version 4.0.5, R Foundation for Statistical Computing). *P* values <.05 were considered statistically significant.

### Ethics Approval

This study has been approved by the Mass General Brigham institutional review board (Protocol #: 2020P002779).

## Results

### Food Timing Assessment

We identified food time stamps and reminder alarms as the 2 features of mobile apps for dietary assessment that are important for accurately capturing food timing (Table 2). Of the 11 mobile apps for dietary assessment reviewed, 8 (73%) recorded food time stamps and 6 (55%) had optional reminder alarms. An important aspect of the food time stamp and reminder alarm features is their ability to be edited by the user. Of the 8 apps that recorded food time stamps, 4 (50%) allowed the user to edit their time stamp on the free version of the app, and 1 (13%) app (Cronometer) had editable time stamps only on the paid version of the app. In addition, all 4 apps that used photo entry included a photo time stamp, and all 3 apps that allowed users to upload photos of their meals included the time stamp of when the photo was initially taken. Of critical importance for food timing research, 2 (18%) of the 11 apps (Bitesnap and FoodView) provided the option to record and edit both food time stamps and reminder alarms.

**Table 2:** Food timing features of the apps selected for review.

App	Record food time stamp	Edit food time stamp	Reminder alarms	Take photo	Photo time stamp	Upload photo	Upload photo time stamp	Edit photo time stamp
Bitesnap	✓	✓	✓	✓	✓	✓	✓	✓
MyFitnessPal			✓					
Cronometer	✓	X^a^						X^a^
MealLogger	✓	✓		✓	✓	✓	✓	✓
FoodView	✓	✓	✓	✓	✓	✓	✓	✓
MyPlate			✓					
myCircadianClock	✓	✓		✓	✓			✓
Macros								
DiaryNutrition	✓							
DietDiary	✓		✓					
FoodDiary	✓		✓					

^a^Feature is available on the paid version.

### Usability

Bitesnap was rated as the most user-friendly app according to the SUS scoring, with a mean SUS score of 91. The least user-friendly app was myCircadianClock, with a mean SUS score of 38.5. Examination of the usability subscores of the 11 apps revealed that Bitesnap had consistently high scores for the positive usability criteria, specifically *app was easy to use* and *felt confident using the system* (Table 3). For the negative usability criteria, Bitesnap had low scores in all categories, indicating high usability. By contrast, myCircadianClock had consistently high scores for the negative usability criteria, specifically *found app* *unnecessarily complex*, *found app very cumbersome to use*, and *needed to learn a lot before using app.*

Most apps scored above average in terms of usability. Only 2 (18%) of the 11 apps (MealLogger and myCircadianClock) were deemed to fall below average owing to scores <68. Of the 11 apps, 4 (36%; Bitesnap, DietDiary, DiaryNutrition, and FoodView) received a SUS score >80.3, which is classified as *excellent* [36], and 5 (45%; MyPlate, FoodDiary, Macros, Cronometer, and MyFitnessPal) received a SUS score between 68 and 80.3, which is classified as *good* [36].

**Table 3:** Positive and negative attributes of usability from the System Usability Scale of the 11 apps selected for evaluation^a^.

Attributes		Bitesnap	MyFitnessPal	Cronometer	MealLogger	FoodView	MyPlate	MyCircadianClock	Macros	DiaryNutrition	DietDiary	FoodDiary
**Positive attributes**												
	Would use app frequently	4.5	2.5	3	1.5	1.5	2.5	1	3.5	2	2	1
	App was easy to use	5	3.5	4.5	4.5	5	4.5	3	4.5	3	5	4
	Various functions were well integrated	4	3.5	3	2.5	4.5	4	3	4.5	5	4	3
	Most would learn to use very quickly	4.5	3	3.5	4	5	4.5	2	4.5	4	5	4
	Felt confident using the system	5	4	4	4	5	5	4	5	5	5	5
**Negative attributes**												
	Found app unnecessarily complex	1	3	2	1.5	1	1.5	4	1.5	1	1	1
	Would need support of technical person to use	1	1	1	1	1	1	1.5	1	1	1	1
	Too much inconsistency	1	1	1.5	1.5	1.5	1	2	1.5	1	1	1
	Found app very cumbersome to use	1	3.5	3	2.5	1	1.5	4.5	1.5	2	1	2
	Needed to learn a lot of things before using app	1.5	1	1	3	1	1	3	1	1	1	1

^a^System Usability Scale scoring on a scale of 1 (strongly disagree) to 5 (strongly agree).

### Data Privacy

This study reviewed the privacy policy specifications of the mobile apps for dietary assessment present on the Apple iOS and Google Play app stores (Table 4). Of the 11 apps reviewed, all 11 (100%) contained a form of privacy policy. A total of 7 (64%) of the 11 apps were found to transmit browser cookies, which are used by sites to track users’ visits and activity. In addition, users’ IP addresses were collected by 8 (73%) of the 11 apps reviewed, and 4 (36%) of the 11 apps collected data on users’ physical location. Of the 11 reviewed apps, only 3 (27%) apps’ privacy policies explicitly described how user data were encrypted. Only 1 (9%) of the 11 apps ensured clear compliance with HIPAA, whereas 7 (64%) of the 11 apps shared data with third parties, and 8 (73%) of the 11 apps used data for research or analytical purposes. In addition, it was found that 2 (18%) of the 11 apps allowed users to use them without having to provide identifiable information.

**Table 4:** Privacy policy specifications of the apps (N=11).

Criteria	Yes, n (%)	No, n (%)	Unspecified, n (%)
Provided privacy policy	11 (100)	0 (0)	0 (0)
Collected identifiable information	9 (82)	2 (18)	0 (0)
HIPAA^a^ compliant	1 (9)	10 (91)	0 (0)
Transmission with third parties	7 (64)	4 (36)	0 (0)
PHI^b^ confidential	7 (64)	0 (0)	4 (36)
Could use app without identifiable data	2 (18)	4 (36)	5 (45)
Able to edit information	1 (9)	3 (27)	7 (64)
Able to delete information	3 (27)	1 (9)	7 (64)
Exportation of the collected data	3 (27)	1 (9)	7 (64)
Data used for research and analytics	8 (73)	1 (9)	2 (18)
App contained links to external sites	3 (27)	1 (9)	7 (64)

^a^HIPAA: Health Insurance Portability and Accountability Act.

^b^PHI: protected health information.

### Nutrient Estimate Differences

#### 4 Sample Items

To assess the accuracy of the apps’ nutrient calculations, a medium-sized orange, 3 Oreo cookies, a peanut butter and jelly sandwich, and a Burger King Whopper were entered into each app and the NDSR database. The caloric and macronutrient estimates from the apps and NDSR are presented in Table 5. For the medium-sized orange, compared with NDSR, the apps had consistent caloric and macronutrient estimates, except for the app Macros, which overestimated calories by 12.1%. Overall, the apps had an average difference of +1.83 kcal (+2.97%) for the orange as compared with NDSR. For Oreo cookies, the nutrient estimates from the apps and NDSR differed by −1.79 kcal (−1.11%). There were no significant differences in the caloric and macronutrient estimates between NDSR and the assessed apps, except for the app Bitesnap, which underestimated calories by 4.7% for the cookies when compared with NDSR. Among the 4 sampled food items, the greatest difference in nutrient estimates was found for the peanut butter and jelly sandwich, which was an average of −24.96 kcal (−6.14%). The smallest caloric difference between the apps and NDSR was found for the Burger King Whopper, which was +0.37 (+0.06%). Although the Burger King Whopper had the smallest average difference across all apps, there was low consistency in nutrient estimates between the apps. For all the food items, the apps were relatively consistent with NDSR in terms of caloric and macronutrient estimates. Cronometer had the least variation for all 4 food items, with an average difference of +0.21 kcal; Bitesnap had the highest, with a difference of −23.79 kcal.

In addition to determining the relative differences between the nutrient estimates for each item, the variance in the nutrient estimates of the apps and NDSR was assessed using paired 2-tailed *t* tests. No significant differences were found in nutrient estimates (calorie, carbohydrate, fat, and protein) between 4 (80%; Bitesnap, Macros, MyPlate, and MyFitnessPal) of the 5 apps that estimated nutrient values and the NDSR calculations. There was a statistically significant difference in carbohydrate estimates (*P=*.02) between Cronometer and the NDSR, but no significant difference was observed for calories, fats, and proteins.

**Table 5:** Macronutrient estimates of food logging apps and Nutrition Data System for Research (NDSR) for the 4 sample food items^a,b^.

Food item			NDSR		Bitesnap		Cronometer		Macros		MyPlate		MyFitnessPal
**Orange (1 medium)^c^**													
	Calories (kcal)	61.6		62.0		62.0		69.0		62.0		62.0	
	Carbohydrates (g)	15.4		15.0		11.9		17.4		15.0		15.4	
	Fat (g)	0.2		0.0		0.2		3.0		0.0		0.2	
	Protein (g)	1.2		1.0		1.2		1.1		1.0		1.2	
**Oreo Cookies (3 cookies)**													
	Calories (kcal)	160.59		153.0		161.0		160.0		160.0		160.0	
	Carbohydrates (g)	24.44		23.0		23.3		25.0		25.0		25.0	
	Fat (g)	6.99		6.0		7.0		7.0		7.0		7.0	
	Protein (g)	1.58		2.0		1.6		1.0		1.0		1.0	
**Peanut butter and jelly sandwich**													
	Calories (kcal)	406.6		323.0		407.0		410.0		378.0		390.0	
	Carbohydrates (g)	50.6		42.0		47.0		48.0		46.0		49.0	
	Fat (g)	18.4		14.0		18.4		18.0		18.0		15.0	
	Protein (g)	12.5		11.0		12.5		15.0		12.0		12.0	
**Burger King Whopper (without cheese)**													
	Calories (kcal)	659.4		655.0		659.0		677.0		678.0		630.0	
	Carbohydrates (g)	50.7		50.0		48.0		54.0		54.0		57.0	
	Fat (g)	38.3		36.0		38.3		37.0		37.0		35.0	
	Protein (g)	26.8		33.0		26.8		31.0		31.0		25.0	

^a^DiaryNutrition, DietDiary, FoodDiary, FoodView, MealLogger, and myCircadianClock did not estimate nutrients.

^b^MealLogger estimated nutrients based on general servings, not specific food items.

^c^Macros had multiple food items for *medium orange*; the first result was used.

#### 3-Day Food Record

The nutrient estimates of the 5 apps that estimated nutrient values (Bitesnap, Cronometer, Macros, MyPlate, and MyFitnessPal) were also evaluated using a sample 3-day food record (Table 6). As shown in Table 7, there was significant variability in the apps’ nutrient estimates, which underestimated calories and macronutrients compared with that of NDSR. The apps reported an average difference of −568 kcal (−11.91%). On average, the apps underestimated carbohydrate, fat, and protein intake by a difference of −27 g (−6.05%), −34 g (−14.8%), −45 g (−17.54%), respectively. Variances in caloric and macronutrient estimates between the apps and NDSR were assessed using paired 2-tailed *t* tests. Significant differences in caloric estimates were found between NDSR and 3 apps, Bitesnap (*P=*.04), Macros (*P=*.04), and MyFitnessPal (*P=*.02), for the 3-day food record. There was no significant difference in carbohydrate estimates between any of the apps and NDSR reference. For estimates of fat intake, 2 apps, Bitesnap (*P=*.03) and MyFitnessPal (*P=*.02), had significant differences compared with the NDSR reference. Finally, for protein estimates, 2 apps, Macros (*P=*.03) and MyPlate (*P=*.04), had significant differences compared with NDSR. Cronometer was the only app with no significant differences in caloric or macronutrient estimates compared with the NDSR reference for the 3-day food record. Finally, we compared the macronutrient estimates of the apps with that of NDSR using 1-way ANOVA; there was no significant difference in calorie or macronutrient estimates between the apps and NDSR.

**Table 6:** Macronutrient estimates of the apps and Nutrition Data System for Research (NDSR) for the 3-day food record.

			NDSR		Bitesnap		Cronometer		Macros		MyPlate		MyFitnessPal
**Day 1**													
	Calories (kcal)	1741		1448		1512		1606		1340		1578	
	Carbohydrates (g)	180		157		150		167		156		185	
	Fat (g)	72		58		53		76		50		62	
	Protein (g)	105		85		96		83		80		86	
**Day 2**													
	Calories (kcal)	1520		1384		1511		1234		1315		1293	
	Carbohydrates (g)	133		140		118		109		129		132	
	Fat (g)	70		60		69		53		58		59	
	Protein (g)	94		72		94		68		77		72	
**Day 3**													
	Calories (kcal)	1509		1308		1464		1320		1343		1353	
	Carbohydrates (g)	128		127		116		133		116		137	
	Fat (g)	88		70		82		78		80		72	
	Protein (g)	56		48		55		43		44		48	
**3-day total**													
	Calories (kcal)	4770		4140		4487		4160		3998		4224	
	Carbohydrates (g)	441		424		384		409		401		454	
	Fat (g)	230		188		204		207		188		193	
	Protein (g)	255		205		245		194		201		206	

**Table 7:** Nutrient estimates of the apps and Nutrition Data System for Research (NDSR) reference for the 3-day food record.

	NDSR	Bitesnap	Cronometer	Macros	MyPlate	MyFitnessPal
Calorie (kcal)	4770	4140	4487	4160	3998	4224
Carbohydrate (g)	441	424	384	409	401	454
Fat (g)	230	188	204	207	188	193
Protein (g)	255	205	245	194	201	206

#### Features of Mobile Apps for Dietary Assessment

Through our evaluation, we identified several features of mobile apps for dietary assessment that are important for researchers to consider when selecting an app for their research purposes. Features evaluated include data input method, data tracked, FCD, and feedback to users (Table 8).

**Table 8:** Features of mobile apps for dietary assessment that are of interest to researchers.

Functions or features		Bitesnap	Cronometer	DiaryNutrition	DietDiary	FoodDiary	FoodView	Macros	MealLogger	MyPlate	myCircadianClock	MyFitnessPal
**Data input method**												
	Text search	✓	✓	✓	✓	✓		✓		✓	✓	✓
	Barcode scanner	✓	✓					✓		✓		✓
	Take photo	✓					✓		✓		✓	✓
	Upload photo	✓					✓		✓			
	Image recognition	✓										✓
	Saved meals	✓	✓	✓	✓						✓	✓
**Data tracked**												
	Calories	✓	✓	✓				✓	✓	✓		✓
	Macronutrients	✓		✓				✓	✓	✓		✓
	Water intake			✓				✓		✓	✓	✓
	Exercise		✓					✓	✓	✓	✓	✓
**Food timing**												
	Meal time stamps	✓	✓	✓	✓	✓	✓		✓		✓	
	Edit meal time stamps	✓					✓		✓		✓	
	Food logging reminders	✓			✓	✓	✓			✓		✓
**Food composition database**												
	Number of food items	≥1300	≥450,000	No information	N/A^a,b^	N/A^b^	N/A^b^	No information	N/A^b^	≥1.3 million	N/A^b^	≥11 million
**Feedback to users**												
	Required nutritional goals		✓	✓				✓	✓	✓	✓	✓
	Text feedback regarding food choice							✓				✓
	Feedback regarding nutritional limits		✓	✓				✓				✓

^a^N/A: not applicable.

^b^MealLogger, FoodView, myCircadianClock, DietDiary, and FoodDiary do not have a food database.

### Data Input Methods

In mobile apps for dietary assessment, users can input dietary intake through several methods, including text search, barcode scanner, saved meals, photo entry, and image recognition. Most apps provided multiple options for inputting food items to make logging easier and more efficient for users (Table 8). Text search was the most common method used to log food, with 9 (82%) of the 11 apps including text search. Barcode scanners were used to select foods in 5 (45%) of the 11 apps. A total of 6 (55%) of the 11 apps allowed users to save frequently consumed meals. Of the 11 apps, 4 (36%) provided users the option to take a photo of their meal, and 3 (27%) allowed users to upload a photo to enter food data. Only 2 (18%) of the 11 apps, Bitesnap and MyFitnessPal, used image recognition technology to identify food options. Of the 11 apps, 2 (18%) allowed users to log food items only through photo input (FoodView and MealLogger). Bitesnap was the only app containing all data input methods (text search, barcode scanner, saved meals, take a photo, upload photo, and image recognition).

### Data Tracked

A full list of the nutrients recorded by each app can be found in Multimedia Appendix 1. A total of 5 (45%) of the 11 apps (DiaryNutrition, DietDiary, FoodDiary, FoodView, and myCircadianClock) recorded no nutritional information. A total of 9% (1/11) of apps (MealLogger) tracked the serving sizes of several food categories but did not calculate nutritional values from the entered food items. MealLogger provided users with visual guides for estimating the serving sizes of food categories, including water, grains, vegetables, fruits, nuts and seeds, legumes, dairy products, meat, seafood, poultry, fats, sugars, supplements, eggs, alcohol, and caffeine. In 1 (9%) of the 11 apps (MyPlate), more nutrients could be tracked in the paid version. Calories, proteins, carbohydrates, and fat were the most popular nutrients tracked, with 6 (55%) of the 11 apps calculating calories and macronutrients. Of the 6 apps that recorded nutrients, the number of nutrients tracked on the free version of the apps ranged from 4 (DiaryNutrition) to 82 (Cronometer).

### Use of FCDs

A total of 6 (55%) of the 11 apps used food databases to record dietary intake. The app with the smallest number of food items in its database was Bitesnap, with just over 1300 items present. By contrast, MyFitnessPal had the highest number of food items, with over 11 million food items recorded. Moreover, 2 (27%) of the 11 apps specified verified food items.

### Feedback to User

**Figure 1:**
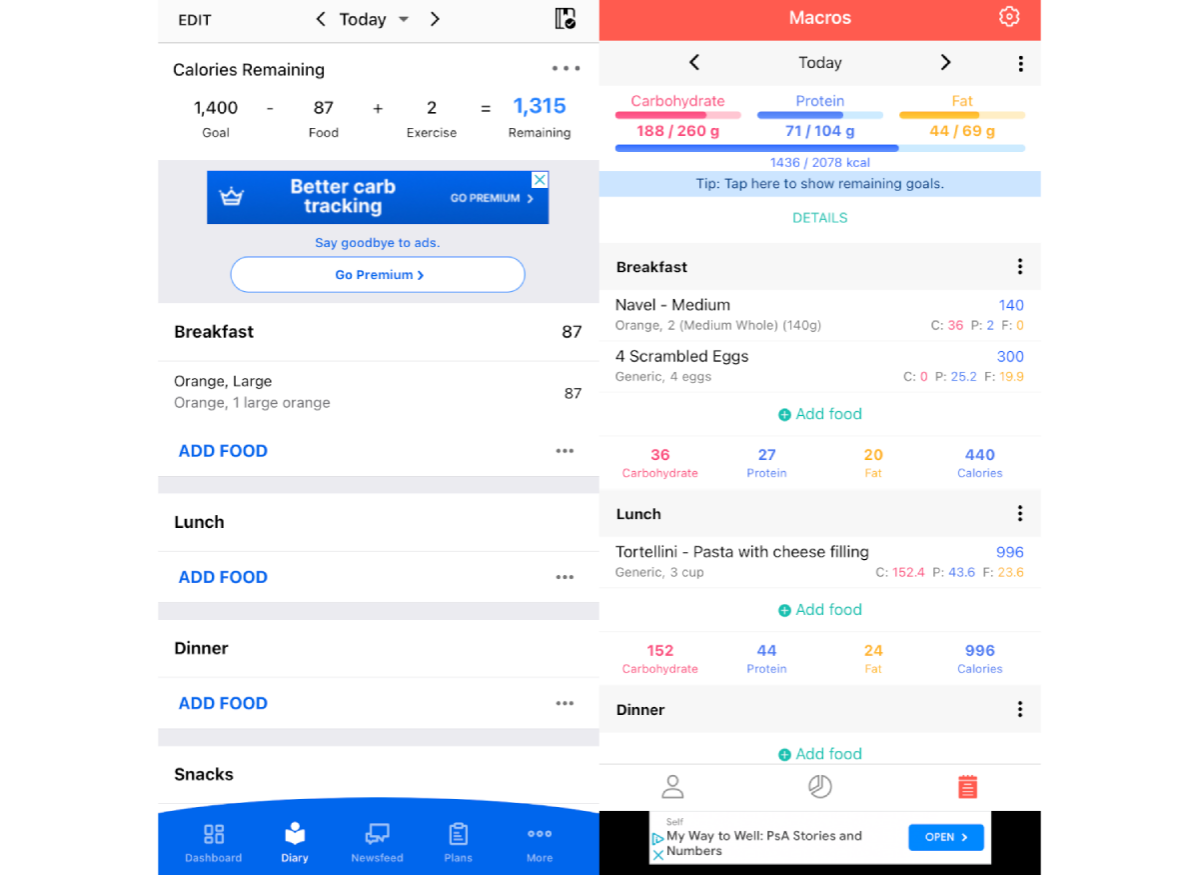
Examples of the text and visual feedback provided to users on MyFitnessPal (left) and Macros (right).

A key feature of certain mobile apps for dietary assessment is the requirement to set nutritional goals, which are generally calculated based on the user’s height, weight, activity level, and health aims. Of the 11 apps, 7 (64%) required users to enter personal information, such as height and weight data, to set nutritional goals, and 6 (55%) of them required a daily caloric limit. In addition to daily nutritional goals, some apps provided feedback to users regarding their dietary intake. Upon reviewing the feedback given by the apps selected for evaluation, we identified two types of feedback: (1) feedback regarding food choice and (2) feedback regarding nutritional limits. Feedback given by the apps was both positive and negative, with positive feedback generally shown in green and negative feedback shown in red. Only 1 (9%) of the 11 apps, MyFitnessPal, provided text feedback regarding food choices. For example, MyFitnessPal shared positive statements such as “this food has lots of vitamin A” and negative feedback informing users that “this food is high in saturated fat.” A total of 5 (45%) of the 11 apps provided feedback regarding personal nutrient limits to users. This included text and visual feedback for logged foods. For example, MyFitnessPal provided negative and positive text feedback to users, such as “your sugar goal is to stay under 54 grams” and “you hit your protein goal for today!” In addition to text statements, several apps used visual feedback, such as turning calorie and macronutrient counters red to indicate that users have exceeded their daily nutritional goals. Figure 1 illustrates examples of text and visual feedback provided by the apps.

## Discussion

### Principal Findings

The mobile apps for dietary assessment selected for review generally had high usability, with 9 (82%) of the 11 apps receiving above-average SUS scores. Usability is an important factor in the consistent and long-term use of health apps. A cross-sectional survey of mobile phone users determined that almost half of the respondents had stopped using an mHealth app, citing the following as primary reasons for stopping app use: high data entry burden, taking too much time to enter data, and apps being confusing [37]. Apps with satisfactory usability will have greater user satisfaction and participant adherence, as participants will be more likely to continue use. In addition, apps with low usability, that is, apps that are confusing or difficult to use, may have lower data accuracy. To evaluate usability for this study, each researcher entered their dietary intake for 2 days and then scored each app using a web-based questionnaire adapted from the SUS. During this evaluation period, we observed that apps with multiple data input methods, such as barcode scanners and photo entry, were more efficient and greatly reduced the time required to log food. Bitesnap was the only app that used every identified data input method (text search, barcode scanner, saved meals, photo entry, and image recognition). Bitesnap was also rated as the app with the highest usability, with a mean SUS score of 91, which may indicate that providing a variety of options for data input makes food logging more efficient and, therefore, increases usability. However, determining what makes an app highly usable remains a challenge for researchers, as usability does not always include evidence-based components. The size of an app’s FCD is also cited as an important factor in the usability of mobile apps for dietary assessment, as apps with more extensive FCDs may decrease participant burden by making food logging less time consuming [22]. However, we observed that greater complexity and volume of the FCD can overwhelm users. The FCDs of the reviewed mobile apps for dietary assessment generally consisted of a combination of the company’s database and foods added by users. Interestingly, Bitesnap had the smallest FCD of all the apps; however, it still received the highest mean SUS score. Furthermore, MyFitnessPal, which possesses the largest FCD of all the apps reviewed, had the third-lowest mean SUS score of all the apps. Although a large FCD can decrease user burden during food logging, based on the SUS scoring, the reviewers found MyFitnessPal unnecessarily complex and very cumbersome to use. Another study surveying the usability of MyFitnessPal raised concerns such as insufficiencies in food database, confusing portion sizes, time-consuming data entry, and poor motivational effects, which may limit the long-term uptake of the app among users in the general population [38]. Although MyFitnessPal had an extensive database, roughly half of the participants found it difficult to navigate the extensive database and noted that food items were not easy to find within the app [38]. 

Although usability is important for user satisfaction and adherence, uncertainties in selecting the appropriate food items and portion sizes present clear concerns for nutrient accuracy. A key characteristic of several of the reviewed mobile apps for dietary assessment is their ability to record the timing of meals. The main features we identified as aiding in accurately assessing food timing are meal time stamps and reminder alarms. Of the 11 apps assessed, 8 (73%) recorded food time stamps, and 6 (55%) allowed users to set reminder alarms. The option to record food time stamps is an important feature that can determine many relevant chrononutrition factors, including fasting duration, dietary patterns, daily food windows, and caloric midpoint. Obtaining this information has the potential to provide substantial insights and greatly inform research on the impact of food timing. Although recording time stamps is incredibly useful, it is especially important that these time stamps be editable. 

Otherwise, incorrect data could lead to inaccurate results. Of the 8 apps that recorded food time stamps, 4 (50%) allowed the user to edit their time stamp on the free version of the app, and 1 (13%) app (Cronometer) had modifiable time stamps only on the paid version of the app. Another important component is the inclusion of a time stamp when entering food data via photos. A total of 2 (18%) of the 11 apps (Bitesnap and FoodView) provided the option to record and edit food time stamps and create reminder alarms. All 4 apps that used photo entry included a photo time stamp, and all 3 apps that allowed users to upload photos of their meals included the time stamp of when the photo was initially taken. Thus, even if an individual does not log their meal manually into the app at the time of consumption, their entry will still reflect an accurate food time, as the app will record the time when the photo of their meal was taken. Accordingly, this feature may also increase the accuracy of food timing assessment. The assessment of food timing can also be improved using reminder alarms; reminding participants who may forget to log their food can greatly improve accuracy and reduce recall errors. In addition, using electronic reminder alarms may increase study validity, as reminder alarms have been shown to improve patient adherence [39]. The presence of these capabilities in mobile apps for dietary assessment is needed to efficiently and accurately assess food timing.

In our evaluation, we assessed the accuracy of the nutrient estimates of the apps in comparison with that of the NDSR reference using 4 sample food items and a 3-day food record. For the 4 sample food items, we observed that the apps were generally consistent with the NDSR reference for calorie, carbohydrate, fat, and protein estimates. The branded items (Oreo cookies and Burger King Whopper) had the smallest difference in caloric estimates between NDSR and the apps. Branded items have nutritional information that is widely available, which likely decreases inaccuracies in measurements. The peanut butter and jelly sandwich had the largest difference in caloric estimates. This discrepancy may be a result of the variability in a generic meal, as it may be composed of several food items that may vary in portion sizes and nutritional content. On the basis of the 3-day food record, we found that the apps consistently underestimated calorie, carbohydrate, fat, and protein intakes. Statistical analysis revealed significant differences in caloric and macronutrient estimates for all the apps compared with NDSR, except for the app Cronometer. Thus, based on the 3-day food record, Cronometer was the most consistent app compared with the NDSR reference. Within the FCDs of 2 (18%) of the 11 apps, verified food items were designated, indicating that the reported nutritional information was reviewed. Interestingly, Cronometer is 1 of the 2 apps to specify verified food items, indicating that verified food items may increase nutrient estimate accuracy. Our findings are consistent with those of previous studies that found mobile apps for dietary assessment to significantly underestimate caloric and nutrient intake [38,40,41]. To the best of our knowledge, most of the selected apps have not been reviewed in the scientific literature or validated for nutrient estimation accuracy. MyFitnessPal has been the subject of several validation studies. Two studies found the app to have relative validity [38,42], whereas another study cautioned about the use of the app owing to significant discrepancies in nutrient estimates [43]. Given these discrepancies, further research is needed to validate the nutrient estimates of mobile apps for dietary assessment.

In evaluating the apps, we found that the primary focus of most apps was on counting calories, often featuring a calorie tracker on the main page of the app, indicating that most apps were developed to be used by the general public as a health tracking or fitness improvement app and not to be used in research or clinical settings without intervention. Only 2 (18%) of the 11 apps, myCircadianClock and Cronometer, stated that scientists and researchers were involved in the development of the app. Several of the apps required users to set daily nutritional goals, specifically daily caloric limits. Apps that require nutritional limits to be set and focus on caloric counting may be an unfavorable choice for use in clinical research, as they may influence participants’ dietary intake and bring about behavioral changes. Apps may also influence the dietary habits of users by providing feedback regarding their intake. Although a study evaluating MyFitnessPal found that most users reported enjoying the feedback provided by the app, research suggests that the feedback statements provided to users may influence them into altering their eating habits [44]. In some cases, it may be possible for researchers to work together with app developers to create a custom version that is more suited for the research goals [45], although cost may be a prohibiting factor.

A systematic review of the privacy policies of mobile apps for dietary assessment is limited [23]. In this study, each policy was thoroughly reviewed according to a set of criteria, including the collection of identifiable information, data encryption, HIPAA compliance, transmission of data to third parties, exportation of the collected data, and app use without the submission of identifiable information. Alarmingly, only 3 (27%) of the 11 apps stated that the data collected were encrypted, and only 2 (67%) of the 3 apps (Bitesnap and myCircadianClock) identified the specific providers. The third app, MyPlate, did not specify their data encryption type but stated that their privacy policies adhered to the European Union–US and European Union–Swiss Privacy Shield Frameworks, which include privacy policies that are stricter than domestic US privacy policy regulations [46]. Transmission with third parties, with reasons being specific to each app, was the most fulfilled criterion, as 7 (64%) of the 11 apps shared the collected data with third parties. Data sharing is done to market products to users, sell user data, and enhance services for users. Unlike the cooperate operator of an app, who have privacy policies, third parties are not required to adhere to the app operator’s privacy policies. Although Apple and Google app stores require app developers to name the third parties with whom user data are shared, no app reviewed in this study identified who the third parties were and how users’ data were used by those parties [47]. Sharing collected data with third parties is a substantial concern, as users have little control over their personal data. The lack of content and breadth in the privacy policies of the apps reviewed indicates that users may be unprepared to make an informed decision and consent to the transmission of their data as per the data privacy and security characteristics set by mobile apps for dietary assessment.

The impact of mobile apps on dietary assessment varies greatly based on app features. Most studies report a strong participant preference [19,45,48] and greater retention rates [49] for app-based dietary assessment compared with website- or paper-based recalls. When evaluating dietary assessment apps, participant and researcher efficiencies vary based on the input method [32], with food photography and automated image scoring leading to the greatest gains in efficiency. Martin et al [50] demonstrated that food photography could result in a smaller error than self-report, and automated image scoring using AI was comparable with or more accurate than trained dieticians in calculating nutrients [51-53]. Keeney et al [34] showed increased efficiency for photo-based versus database entry caloric analysis (35 min/wk vs 85-90 min/wk). A current challenge for photo-based assessment apps lies in the ability of AI to accurately recognize and process food photography and videography, with Shen et al [51] reporting 78% to 92% accuracy when used in a curated database versus 0.26% to 0.49% accuracy when used in the general population. More refinement by training models across a broader collection of varied foods is needed to increase the utility of AI in dietary assessment and create true gains in efficiency for both participants and researchers.

### Comparison With Prior Work

Shinozaki et al [54] published research evaluating the accuracy of energy and nutrient estimates by mobile apps in Japan. One of the apps we assessed overlapped with their assessment (MyFitnessPal). Ferrara et al [55] and Chen et al [56] also compared the usability (using the SUS scoring) and accuracy of energy and nutrient estimates of mobile food logging apps; however, they performed their analysis in the context of behavior change theory and weight loss, not in the context of use of the apps in the clinical setting. To the best of our knowledge, no assessment of the capability of mobile apps to capture key food timing details has been performed.

### Limitations

This study has several limitations that should be addressed. The usability of the apps was reviewed by 7 researchers with relatively high digital literacy. Thus, their assessment of the usability of the mobile apps for dietary assessment may not be generalizable to a larger population. In addition, for the usability scoring, 4 of the reviewers used the iOS version of the apps, and 3 reviewers used the Android version of the apps. However, we did assess the differences in the SUS scoring between the Android and iOS versions of the apps. Another limitation of this evaluation is that the food items used to assess the nutrient estimate accuracy of the apps may not be representative of the dietary intake of the larger population, given that the purpose of our 3-day food record was to test consistency between the app and the registered dietitian nutritionist. Most often, the general population consumes mixed meals that are not branded or adds additional food items to meals, which were not accounted for in this study. To complete this analysis, food items were entered into each app by a single researcher and not verified by a second reviewer, which may have resulted in input errors. In addition, to compare the nutrient estimates of the apps and NDSR, food items were entered into the apps by 1 researcher, whereas food items were entered into NDSR by a registered dietitian nutritionist, which could have resulted in variation. Of the 11 apps evaluated, 6 (55%) were developed in the United States, which may cause the nutrient estimates to not be applicable to other countries. Finally, although several of the apps used multiple data input methods, such as a barcode scanner or image recognition, the accuracy of nutrient estimates was assessed solely using text search, as it was the only method present in every app selected. The evaluation of nutrient accuracy using other data input methods should be explored in future studies.

### Conclusions

Although there are many food logging apps on the market that are available to consumers, not all of them fit the needs of clinical research for use in studies of circadian rhythms and food timing in populations with shifted mealtimes. We took some of the most consumer-friendly and research-viable apps and compared them against one another. Overall, photo input can increase the accuracy of the time stamp by indicating when the food was consumed because it does not rely on an individual’s memory recall. Throughout the 3-day food record, the Cronometer app was the most accurate app. Overall, the Bitesnap app was the most flexible based on user preferences, offering users all ways of inputting their meal (photo, text, etc), and was ranked highest in usability according to the participants. Although our pool of reviewers was small and had a relatively high digital literacy, our study generated useful preliminary data on the accuracy and usability of the reviewed apps, keeping an eye on the integration of the food timing data into dietary app data collection.

## Appendix

Multimedia Appendix 1 Nutrients recorded by each app.

## Abbreviations

24HR: 24-hour dietary recall
AI: artificial intelligence
FCD: food composition database
FD: food diary
HIPAA: Health Insurance Portability and Accountability Act
mHealth: mobile health
NDSR: Nutrition Data System for Research
SUS: System Usability Scale
